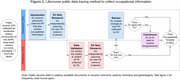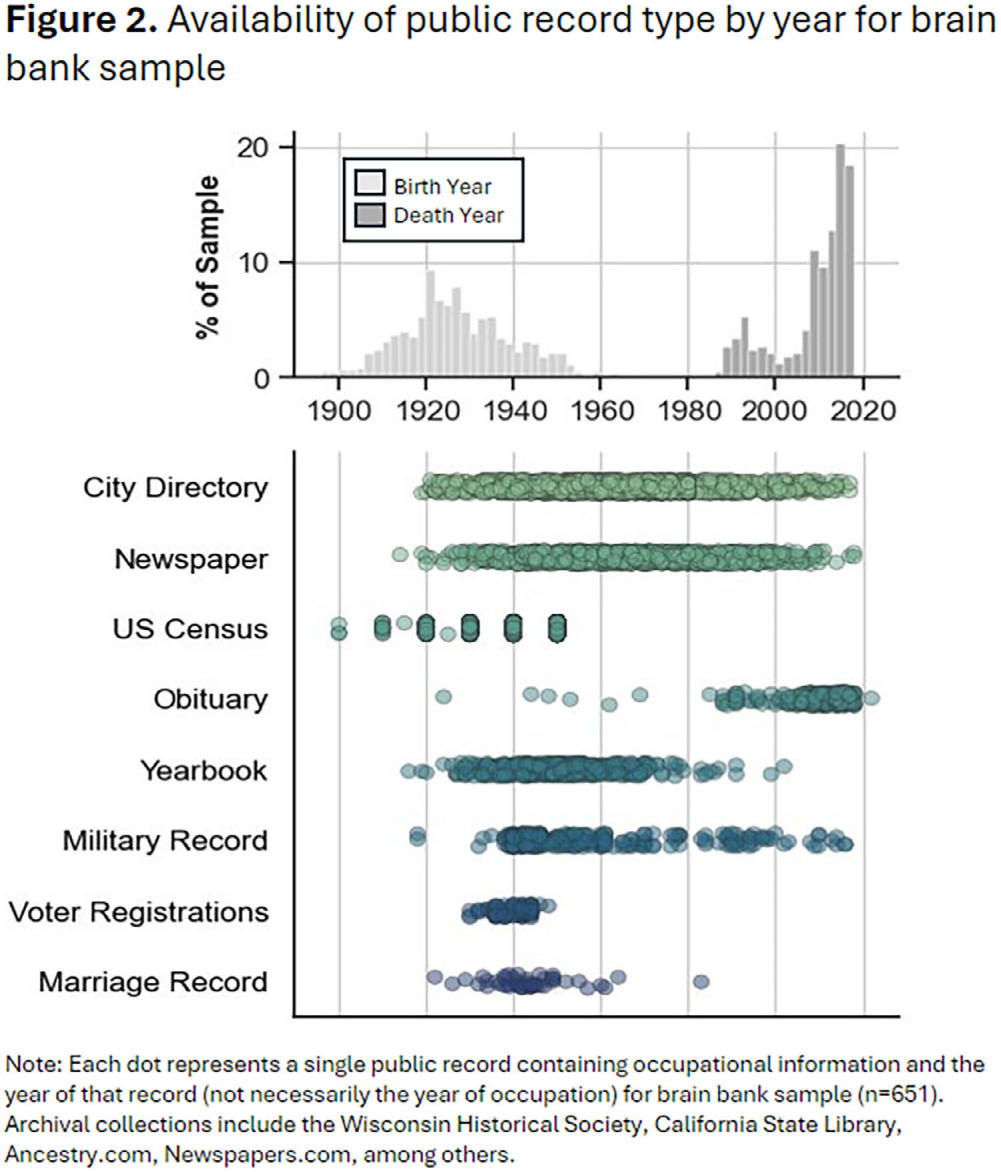# Public data tracing: A novel method for retrospectively collecting occupational data for brain bank research donors

**DOI:** 10.1002/alz.090940

**Published:** 2025-01-09

**Authors:** Rachel C. Otte, Lauren Etter, Elliot I. Surovell, Sarah Lim, Aly Pfaff, Amanda DeWitt, Jessica Sharrow, Shahriar Salamat, Megan L. Zuelsdorff, Robert A. Rissman, Barbara B. Bendlin, Amy J.H. Kind, W. Ryan Powell

**Affiliations:** ^1^ Center for Health Disparities Research, University of Wisconsin School of Medicine and Public Health, Madison, WI USA; ^2^ Department of Neurological Surgery, University of Wisconsin School of Medicine and Public Health, Madison, WI USA; ^3^ Department of Pathology and Laboratory Medicine, University of Wisconsin School of Medicine and Public Health, Madison, WI USA; ^4^ Wisconsin Alzheimer’s Disease Research Center, University of Wisconsin School of Medicine and Public Health, Madison, WI USA; ^5^ University of Wisconsin School of Nursing, Madison, WI USA; ^6^ Alzheimer’s Therapeutic Research Institute, Keck School of Medicine, University of Southern California, San Diego, CA USA; ^7^ Veterans Affairs San Diego Healthcare System, San Diego, CA USA; ^8^ Wisconsin Alzheimer’s Institute, University of Wisconsin School of Medicine and Public Health, Madison, WI USA; ^9^ Department of Medicine, Geriatrics Division, University of Wisconsin School of Medicine and Public Health, Madison, WI USA

## Abstract

**Background:**

Working conditions and contexts may influence the development of Alzheimer’s Disease and related dementias (ADRD), exposing individuals to modifiable risk factors across their life. Measurement of ADRD pathology at autopsy provides a gold standard outcome for evaluating the effects of lifetime exposures, but approaches to quantify ante‐mortem work exposures are limited. Here we describe a new method to retrospectively capture occupational histories by systematically extracting occupational information using archival public records— i.e., public data tracing—and provide preliminary feasibility for ongoing data collection for two ADRC brain bank cohorts.

**Methods:**

Our sample includes individuals donating to two ADRC brain banks between 1986‐2018 who had residential histories previously constructed and are part of ongoing efforts to build occupational histories (n = 651 of a planned 1154). They had a mean age at death of 81.7 (SD = 10.3) years, were 51.9% (n = 338) female, and had a median year of birth = 1926 and median year of death = 2012. Quantifying occupational exposure entails systematic searches of archival collections available in‐person and online (e.g., Ancestry.com) to locate records (Figure 1). Two researchers independently verified the identity and content of public records using the Genealogical Proof Standard. Data collection occurred from June‐December 2023.

**Results:**

We identified an average of 10.7 (SE = 0.28) records with occupational information per donor with an average 1.29 (SE = 0.02) hours spent extracting and validating each donor’s occupational history. The most common public record types included: federal censuses (≥1 record for 70.5% of donors), city directories (70.2%), obituaries (62.6%), newspapers (58.3%), yearbooks (40.1%), military records (27.7%), marriage records (6.9%), and voter registrations (6.2%), which varied by time (Figure 2). In addition, we found the number of public records varied by sex, with more records containing occupational information identified for males (14.2 average records) compared to females (7.2).

**Conclusions:**

Preliminary findings suggest that applying public data tracing methods to collect occupational information is feasible. There are both limitations and opportunities, particularly as public record availability seems to vary by time (both calendar and lifecourse) and donor sex. This new method quantifies occupational exposures over the lifecourse, creating new opportunities to study the relationship between work and ADRD brain health.